# Multiquanta flux jumps in superconducting fractal

**DOI:** 10.1038/s41598-023-39733-y

**Published:** 2023-08-03

**Authors:** Vitalii K. Vlasko-Vlasov, Ralu Divan, Daniel Rosenmann, Ulrich Welp, Andreas Glatz, Wai-Kwong Kwok

**Affiliations:** 1 Materials Science Division, Argonne National Laboratory, Argonne, IL 60439 USA; 2 Center for Nanoscale Materials, Argonne National Laboratory, Argonne, IL 60439 USA; 3 Department of Physics, Northern Illinois University, DeKalb, IL 60115 USA

**Keywords:** Materials science, Condensed-matter physics, Materials for devices

## Abstract

We study the magnetic field response of millimeter scale fractal Sierpinski gaskets (SG) assembled of superconducting equilateral triangular patches. Directly imaged quantitative induction maps reveal hierarchical periodic filling of enclosed void areas with multiquanta magnetic flux, which jumps inside the voids in repeating bundles of individual flux quanta Φ_0_. The number N_s_ of entering flux quanta in different triangular voids of the SG is proportional to the linear size *s* of the void, while the field periodicity of flux jumps varies as 1/*s*. We explain this behavior by modeling the triangular voids in the SG with effective superconducting rings and by calculating their response following the London analysis of persistent currents, J_s_, induced by the applied field H_*a*_ and by the entering flux. With changing H_*a*_, J_s_ reaches a critical value in the vertex joints that connect the triangular superconducting patches and allows the giant flux jumps into the SG voids through phase slips or multiple Abrikosov vortex transfer across the vertices. The unique flux behavior in superconducting SG patterns, may be used to design tunable low-loss resonators with multi-line high-frequency spectrum for microwave technologies.

## Introduction

Fractal structures with self-similar repetition of topologically identical features at diminishing length scales are universally found in nature (from plant leaves and seashells to blood vessels and neural networks^1,2^). They are frequently reported in materials studies (from molecular assemblies^3^ to domain structures in quantum magnets^4^), and are often employed in technological devices (from compact antenna designs^5^ to efficient heat exchangers^6^ and advanced load supports^7^).

**Figure 1:**
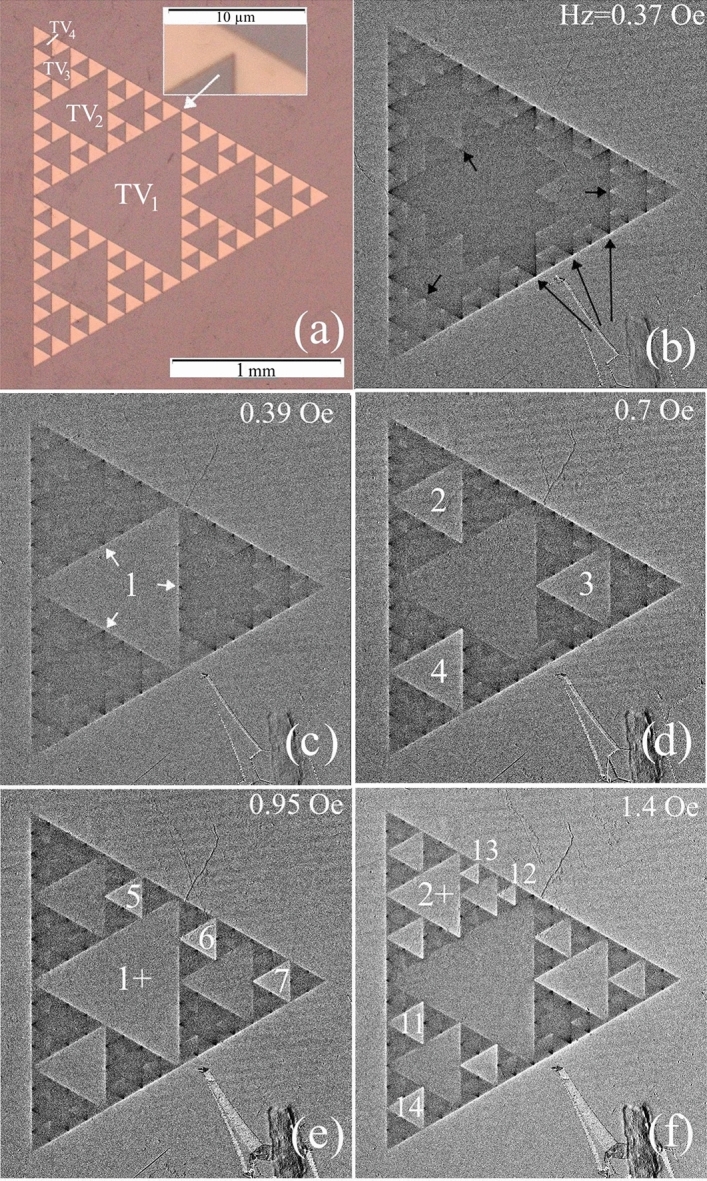
(**a**) Picture of a 3^d^-order Sierpinski gasket (SG) consisting of 100 nm thick Nb film equilateral triangular patches (bright) with triangular voids (dark) of proportionally decreasing size marked as TV_1_ (1 mm side) to TV_4_ (125 µm side). The insert shows the expanded view of 1 µm bridges between the Nb patches. (**b**–**f**) Magneto-optical images of a few successive flux jumps in triangular voids of the SG with increasing magnetic field H_z_^*a*^ applied perpendicular to the sample plane at T = 3.5 K. The strength of contrast in the MO image inside the TVs and at their boundaries corresponds to the strength of the normal field induction B_z_. Short arrows in (**b**) point to the enhanced positive B_z_ (B↑↑H_z_^*a*^, bright) at the vertices of the internal TVs caused by the distributed Meissner currents in the SG. Long arrows in (**b**) show increased negative B_z_ (B↓↑H_z_^*a*^, dark) near the vertices of TVs abutting the sample’s edge. Bright contrast lines along the outer periphery of the sample reveal the enhanced edge field due to the screening effect similar to that in a continuous SC triangle. Consecutive instant flux jumps in the TVs begin with the largest central TV_1_ and proceed to smaller TVs. Numbers in (**b**–**f**), indicate the sequence of flux filling order of the TVs. The order of flux filling from large to small TVs is sometimes disrupted by early flux entry into the smallest TVs. Likewise, with increasing field, periodic flux entry into the largest TV may repeat several times before the flux entry occurs in smaller TVs (see the second round of jumps into TV_1_ and TV_2_ marked as 1 + in (**e**), and 2 + in (**f**)).

In particular, Sierpinski gaskets (SG), formed by triangles of progressively decreasing size (the fractal recursive rule is illustrated in Fig. 1) offer unique electromagnetic response desirable for advanced microwave applications^8,9^. Their parameters essentially can be improved using loss-less superconducting materials, in which case the SG becomes a multiply connected superconductor (SC) with different scale array of voids. Prior studies of SGs comprised of SC wires or wires with Josephson Junctions which showed distinct hierarchical and repetitive changes in resistivity and inductance of the samples in applied fields near the SC transition temperature (T_c_)^10–15^. These samples were lattices of Sierpinski gaskets up to 6th order with elementary triangles of submicron or a few micron size. In small applied magnetic fields, it was possible to successively fill different triangular subsets composing the SG with individual magnetic flux quanta, Φ_0_ = πħ/e. The hierarchy of flux filling, resulting in sharp changes of T_c_ or inductance of the SG arrays, followed digital flux quantization rules, NΦ_0_ → (N ± 1)Φ_0_, commonly reported for multiply-connected superconductors, with specifics imposed by the fractal pattern geometry. For experiments close to T_c_, the data analysis is simplified due to negligible Meissner screening, resulting in homogeneous magnetic field distribution (see^10–16^ and refs. there). However, at low temperatures (T), where losses are desirably minimized, the screening effects become important and the magnetic field is modified by SC persistent currents. Moreover, due to increased critical currents at low T, the flux entry into the samples is strongly delayed and may depend on the dynamics of phase slips or entry of Abrikosov vortices which can transfer single or multiple flux quanta into the voids inside the superconductor.

In this work we directly image the magnetic flux entry in a millimeter sized Sierpinski gaskets comprised of equilateral superconducting triangles encasing sequentially decreasing triangular voids. We find that at temperatures well below T_c_, flux behavior is characterized by consistent well-structured hierarchical succession of multiquanta flux jumps. The flux entry is qualitatively similar to single-quantum-flux jumps observed in microscopic SG patterns at T ~ T_c_. However, unlike such single-Φ_0_ representative of Little-Parks oscillations, in our samples at T ~ T_c_/2 the repeating flux jumps consist of thousands of Φ_0_, depending on the size of the triangular voids in the SG structure. Also, the imaged inhomogeneous field distributions induced by SC persistent currents affected by the flux jumps, reveal interactions between different flux cells, sometimes resulting in combined positive and negative jumps in the neighboring voids of the SG.

We envision that our superconducting SG patterns, where changes of inductance, caused by the redistribution of currents due to orderly flux jumps controlled by small magnetic fields, can shift the SG eigen-frequencies, and hence can be used as tunable low-loss multiline resonators for quantum IT devices and sensors. In turn, a wide set of possible combinations of diverse N_s_Φ_0_ flux bits trapped in the 2D array of different SG triangular voids could be employed for advanced digital recording.

## Experiment

We used the magneto-optic indicator technique (MOI)^17^ to image the magnetic flux penetration in an equilateral triangular SG structure with maximum triangle side of 2 mm fabricated from a 100 nm niobium film with superconducting (SC) transition temperature T_c_ = 8.75 K, grown by high-vacuum magnetron sputtering. A Sierpinski gasket obtained after successive removal of progressively decreasing triangular areas while leaving narrow 1 µm bridges between vertices of the remaining triangular SC patches is shown in Fig. 1. In SG structures made of thin wires, the 0-*order* gasket is a simple equilateral triangle and the order increases upon successive addition of wires connecting the centers of the larger triangle sides. In our case the 0-*order* gasket corresponds to three triangular patches surrounding the central triangular void. The equivalence with the 0-*order* wire gasket is that we similarly begin with one hole in the SC structure. Below we present the main results for our highest 3^d^-order SG pattern (Fig. 1a), which is formed after an eightfold reduction of the largest triangular void, yielding the smallest triangles with 125 µm sides.

The macroscopic magnetic response of the samples in a magnetic field was measured using SQUID magnetometry, and the flux distributions at T < T_c_ were observed using MOI. The samples were mounted on a cold finger of a commercial Montana cryostat and covered by an indicator film with a large Verdet constant to spatially visualize the normal magnetic field at the sample surface, B_z_(x,y), in polarized light. Careful calibration of image intensity versus applied normal field H_z_^a^ at T slightly above T_c_ allows accurate quantitative assessment of induction distributions in the sample.

We start with demonstration of the magnetic flux entry in our 3^d^-order SG structure using a set of MOI pictures obtained by gradually increasing the applied field, H_z_^a^, where the image intensity corresponds to the local strength of B_z_. Quantitative changes of local B_z_ within various triangles in our SG samples will be presented below as B_z_(H_z_^a^) plots.

Figure 1b–f show successive evolution of the B_z_ map in the sample with increasing H_z_^a^ in steps of ΔH_z_ ~ 0.03 Oe at T = 3.5 K. At this temperature, the magnetic field ≲ 0.4 Oe is mostly screened from the entire sample by Meissner currents J_M_ (Fig. 1b). B_z_ increases only outside the peripheral of the sample as expected in a continuous SC triangle. However, peculiar weak B_z_ features concurrently appear inside the sample along the contours of all triangular voids (TVs). Specifically, small *negative* B_z_**↓↑**H_z_^a^ (dark contrast as opposed to bright B_z_**↑↑**H_z_^a^) is observed at the sides and in the vertices of the TVs located adjacent to the outer-edges of the SG sample as marked by longer arrows in Fig. 1a (see also enlarged images in Fig. A1 of the Supporting Info).

**Figure 2:**
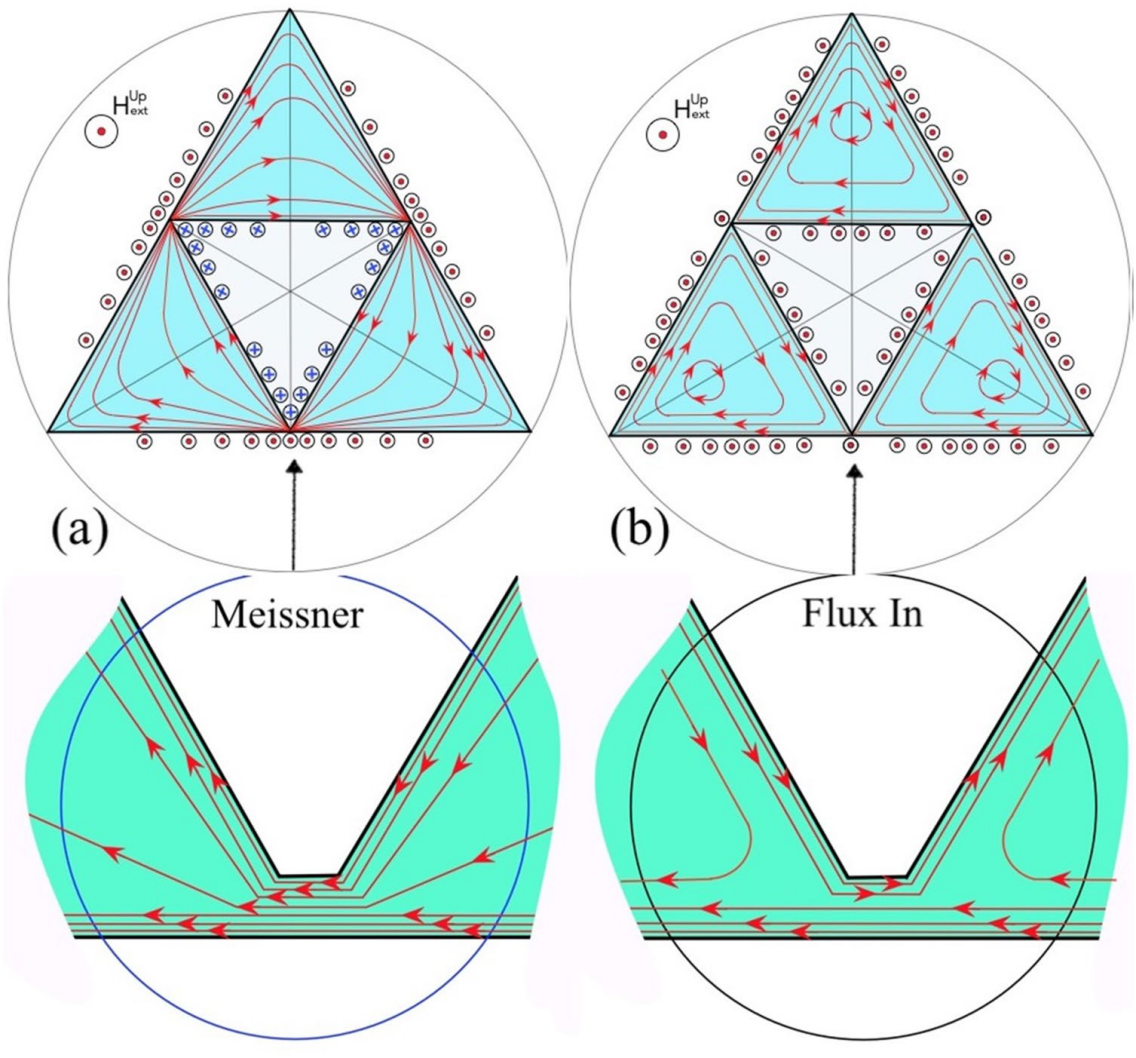
Sketch of the current trajectories (red lines with arrows) and current induced fields (small circles with red dots and blue crosses for B_z_ pointing Up and Down respectively) in 0th-order superconducting Sierpinski gasket in the Meissner state (**a**) and after the magnetic flux jump inside the triangular void (**b**). Bottom panels illustrate the current pattern around the narrow bridges linking the triangular SC patches in the SG.

Another peculiarity, a slightly enhanced positive B_z_ (bright contrast), emanates from the vertices of the smallest voids, TV_4_, at the sides of larger internal TVs as marked by short arrows in Fig. 1b. These features appear due to the unidirectional screening currents J_M_ distributed over triangular patches of our SG. In the Meissner state, in a multiply connected SC structure with a single hole, such as a ring, the screening current, J_M_, is concentrated near the inner and outer ring edges but has the same polarity across the ring’s width^18–21^. As a result, the applied field is enhanced at the outer ring edge, while the local negative (opposite to H_z_^a^) field appears at the inner edge (see Fig. A2 in Supporting Info). Further inside the hole, the field reverses sign again and a small positive B_z_ forms in the center, but the total flux over the entire ring area is smaller than Φ_0_ and the ring remains in the Meissner state. The sketch of the J_M_ distribution in SG structures following the above scenario, which explains details of the Meissner B_z_ map observed in our samples, is shown in Fig. 2a. The time dependent Ginzburg–Landau (TDGL) solution for the current distributions in the SG is presented in Fig. A3 of the Supporting Info.

With slow increase of H_z_^a^, the above described features remain qualitatively unchanged although their contrast slightly increases. Then, at H_z_^a^ ~ 0.4 Oe the magnetic flux suddenly jumps into the large central TV_1_ (Fig. 1c) where the enhanced bright contrast at the edges signify B_z_ > H_z_^a^. The B_z_ contrast at the sides of TV_1_ changes from dark to bright, indicating the inversion of the current direction near these edges. Consequently, the local SC current here, responds to the injected flux Φ_1_ instead of just screening the applied field H_z_^a^. Appropriate sketch of the changed current distribution is shown in Fig. 2b (the TDGL solution is presented in right panel of Fig. A3 of Supporting Info). The total flux in the central TV_1_, estimated using measured B_z_ in the triangle at H_z_^a^ ~ 0.4 Oe and the triangle area, is ΔΦ_1_ ~ 6600 Φ_0_ (see details below).

**Figure 3:**
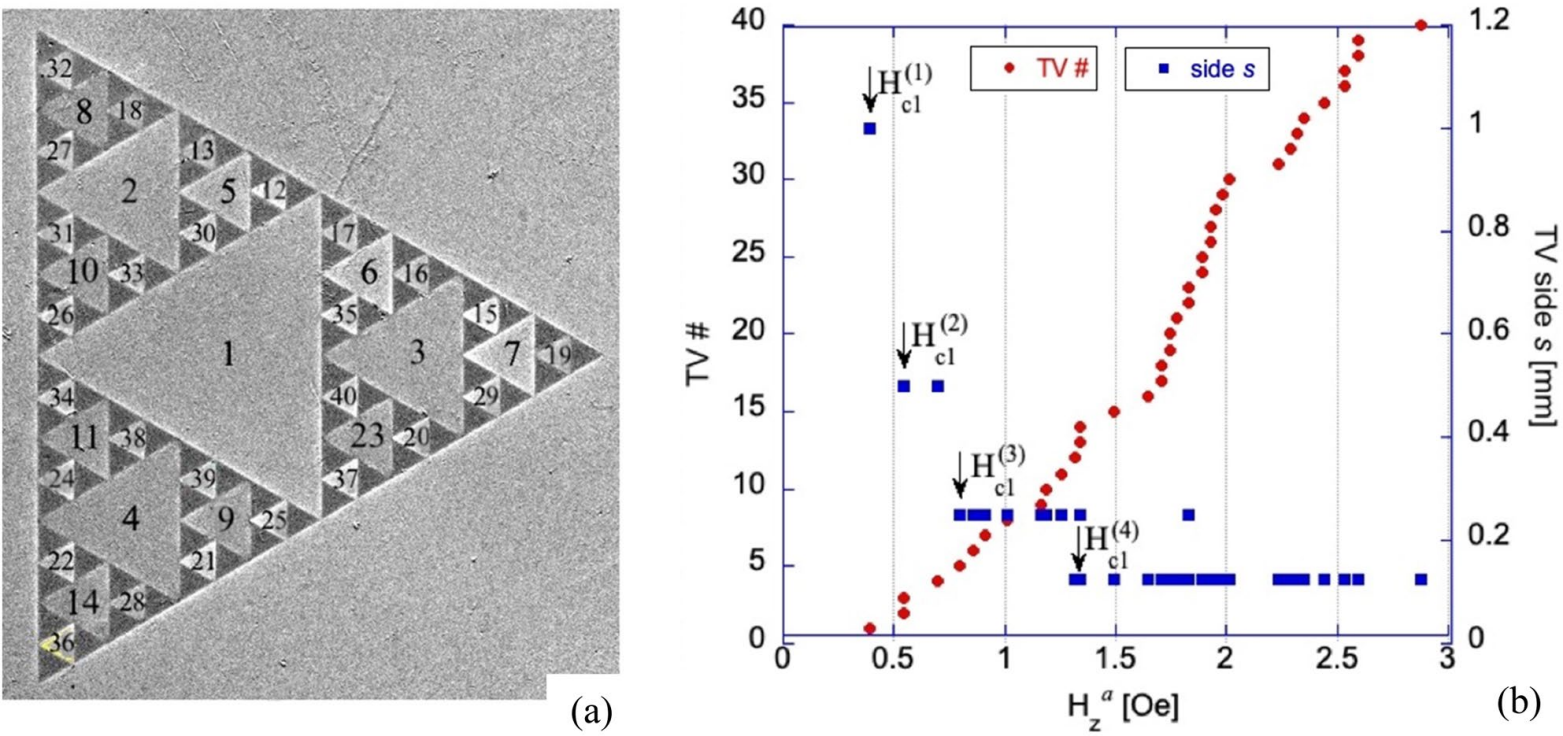
(**a**) Numerical order of the sequence of first flux jumps into the triangular voids (TVs) of the Sierpinski gasket with increasing applied field H_z_^*a*^. (**b**) Dependence of the flux filling sequence (from TV#1 to TV#40, left ordinate) on the magnetic field H_z_^*a*^ (red dots). Blue squares show the side length *s* of the appropriate TVs (right ordinate) indicating the major tendency of the flux entry, from the largest to the smallest triangles. Arrows mark fields of the first flux entry in successively smaller triangular voids, from H_c1_^(1)^ for *s* = 1 mm to H_c1_^(4)^ for *s* = 125 µm.

With further increasing field, the jump-wise flux filling occurs in the next smaller sized, TV_2_ (*s* = 0.5 mm) marked as 2–3–4 in Fig. 1d. TV_2_-#2 and -#3 are filled simultaneously and TV_2_-#4 is filled at slightly larger H_z_^a^. The abrupt flux jumps are accompanied by the dark-to-bright reversal of contrast at the TV_2_ edges, as described above for TV_1_. Following the jump, the field in TV_2_ is higher than B_z_ in TV_1_ but the flux change is smaller (ΔΦ_2_ ~ 2600 Φ_0_) due to the smaller triangle area.

After the flux enters the set of TV_2_, the next smaller TV_3_ voids (#5, 6, 7…, *s* = 0.25 mm) begin to fill with magnetic flux at H_z_^a^ > 0.8 Oe (Fig. 1e). Flux jumps in voids of TV_3_-set progress at small field intervals, sometimes in pairs of TVs, but not simultaneously in all TV_3_ voids. In some cases, during the process of filling the smaller TVs, the additional flux jumps occur in larger TVs where the total flux is repeatedly increased by the same value of ΔΦ_i_ (see TV_1_ after the 2nd jump marked “1 + ” in Fig. 1e, and “2 + ” for TV_2_ in Fig. 1f). With further increasing field, at H_z_^a^ > 1.32 Oe, slightly before all TV_3_ voids are filled, the next smaller set of voids (TV_4_, *s* = 0.125 mm, #12_,_ #13 and so on) begin filling (Fig. 1f). In some cases, they fill in pairs with TVs of the same or different size*,* and the succession of appropriate filling steps is intermittent with incremental ΔΦ_i_ jumps in larger TVs.

**Figure 4:**
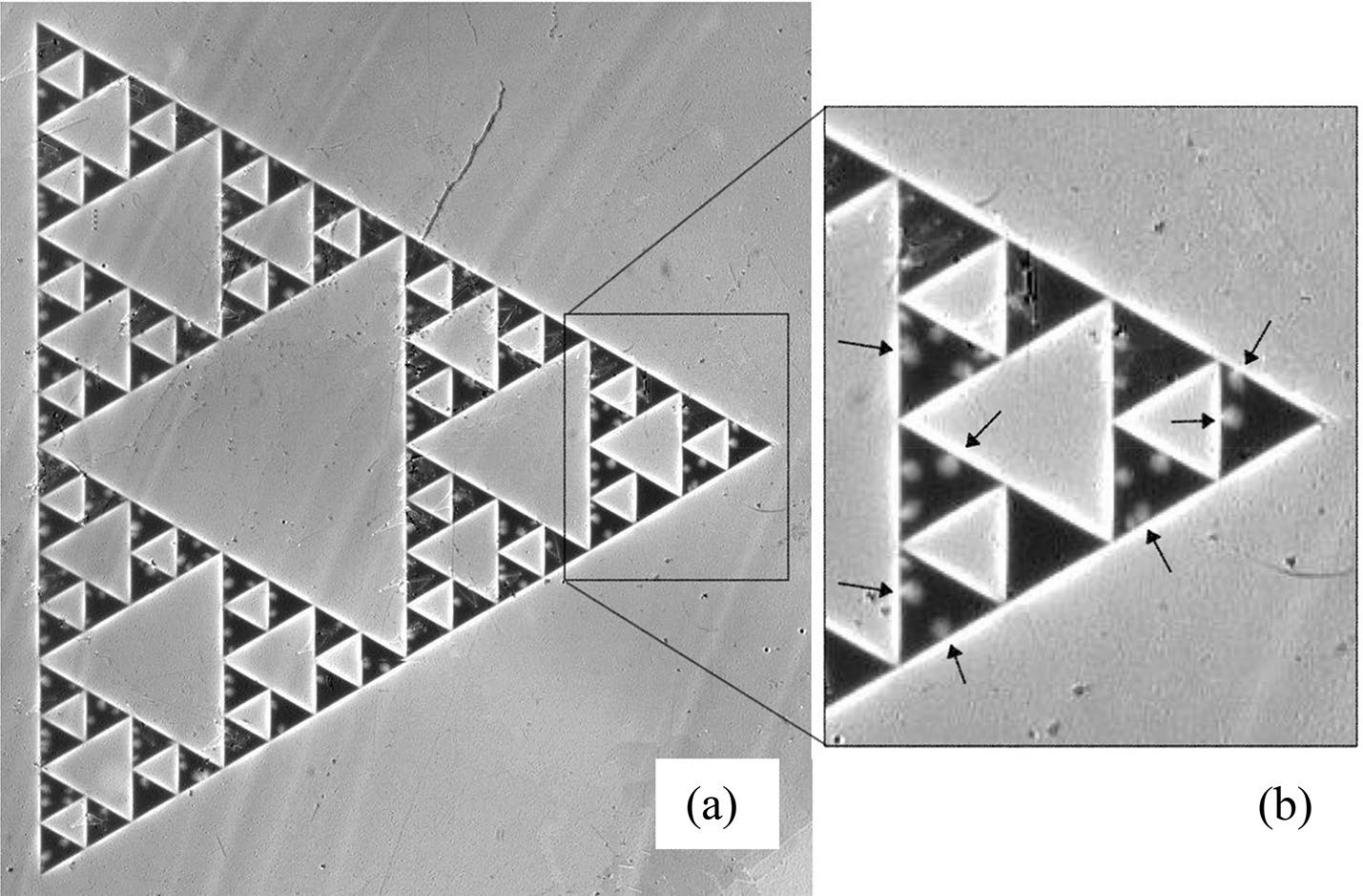
(**a**) Entry of Abrikosov vortices into the superconducting areas of the Sierpinski gasket. All the triangular voids are filled with magnetic flux (bright contrast) and vortices start penetrating the flux-free (dark) Nb triangles. MOI taken at T = 3.5 K, H_z_^*a*^ = 32.7 Oe. Right panel (**b**) shows the expanded view of the boxed fragment on the left. Arrows point to flux balloons of multiple vortices penetrating from all edges of the superconducting triangles.

Finally, following multiple repeated flux jumps in larger voids, all 40 TVs in the SG are filled with flux at H_z_^ap^ ~ 2.9 Oe. The sequence of filling presented in Fig. 3 shows how the *first flux jump* occurs in each of the TV_i_ upon increasing H_z_^a^. Clearly, the field of the initial flux jump, and the range of fields required for filling all the TV_i_ of the same size *s*, increases with decreasing *s*. With further increase in H_z_^a^, additional flux jumps repeat periodically in all the TV_i_. Eventually, after the triangular voids are filled, Abrikosov vortices start entering the SC patches at relatively large fields H_z_^a^ > 22 Oe (Fig. 4).

**Figure 5:**
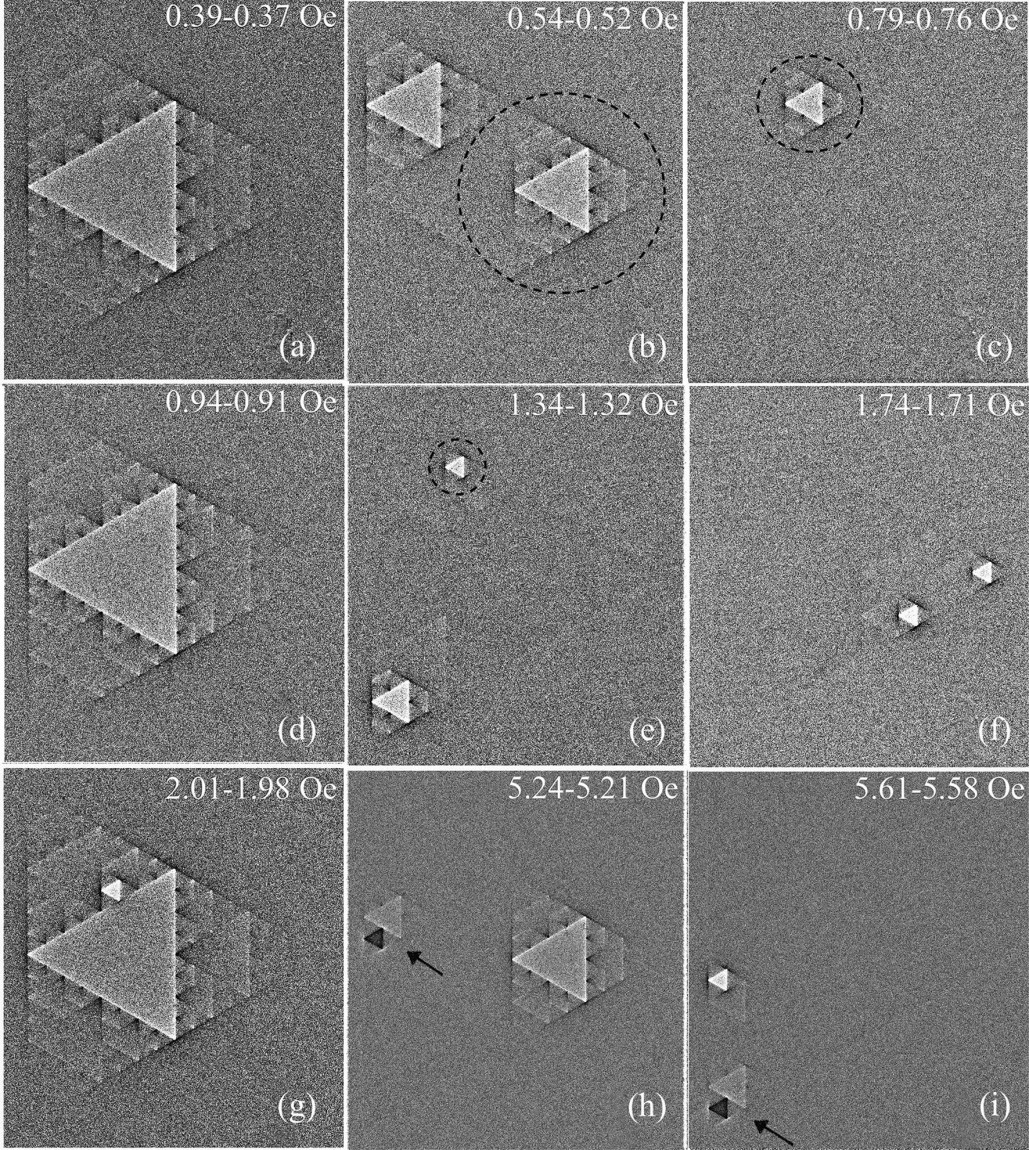
Difference images, obtained by subtraction of B_z_-maps preceding and following the flux jump in different sub-SGs, revealing the abrupt change of the sub-SG current flow pattern. In (**a**), the enhanced bright contrast (ΔB_z_ > 0) along the edges of the central triangular void (TV_1_) corresponds to the inversion of the screening currents J_M_ near these edges to support the trapped flux in TV_1_. In turn, the stronger dark contrast along the boundaries of the entire sample (ΔB_z_ < 0) shows a noticeable drop in J_M_ there. Qualitatively similar difference patterns are observed after flux jumps in smaller TV_i_s. They show ΔB_z_ changes well localized within appropriate lower order sub-SG_i_ due to the current inversion at the TV_i_ edges and decreased currents at the sub-SG_i_ boundaries. In panels (**b**), (**c**), and (**e**) the 2^d^, 1st, and 0-order sub-SG_i_s are encircled by dashes. Similar ΔB_z_ changes repeat after second and further jumps in the same TV (compare e.g. (**a**) and (**d**) or (**b**) and (**h**)). The distributed Meissner currents, which spread over the sub-SG_i_ area define slight increase or decrease of B_z_ at the vertices and along the sides of smaller TVs inside the sub-SG_i_ in all pictures. More complex patterns appear during rare negative jumps (dark triangles in (**h**)–(**i**) pointed by arrows) which are accompanied by a partial positive jump in neighboring TVs.

Important details of the changing current patterns during the flux jumps in our samples are revealed by difference images presented in Fig. 5. They are obtained by subtraction of sequential B_z_ images before and after the flux jump and represent increments of ΔB_z_(x,y) = B_z_(H_z_^*a*^ + 0.03 Oe)-B_z_(H_z_^*a*^) corresponding to appropriate changes of the currents ΔJ (x,y) during the jump. Figure 5 shows that the flux jumps ΔΦ_i_ in any sized TV_i_ yield qualitatively the same picture of a homogeneous ΔB_z_ over the main TV_i_ area with enhanced positive ΔB_z_ at the TV_i_ periphery. Pronounced contrasting features emerge in three neighboring triangular regions around the TV_i_. They have the same size as TV_i_, but contain smaller triangular voids surrounded by SC patches. Together with the central TV_i_ where the flux jump occurred (brightest contrast), these neighbors make up the Sierpinski sub-gaskets (sub-SG) of a lower order. Such smaller sized sub-SGs of order 2, 1 and 0 are isolated with dashes in Fig. 5b,c,e, respectively.

The ΔB_z_ patterns show that after the flux jumps into a TV_i_, the screening currents J_M_ in the SC patches surrounding the TV_i_ are inverted along the edges of the TV_i_ and also along the sides of the smaller TVs making up the sub-SG_i_ structure. At the same time, J_M_ is noticeably reduced at the sub-SG_i_’s outer boundary. The picture corresponds to the changes from pattern (a) to (b) in current distributions sketched in Fig. 2. Note that for successive jumps in the same TV taking place with increasing field, the difference pattern remains the same (compare Fig. 5a and d) confirming the replicability of the repeated flux jump cycle. Also, at H_z_^a^ beyond the jump field, the sub-SG_i_ B_z_-maps (not shown) restore the pre-jump features qualitatively similar to Fig. 1b and reveal the reemerging screening current distribution akin to the Meissner state pattern sketched in Fig. 2a.

In addition to successive flux jumps with increasing H_z_^a^, at larger fields we observe unexpected local *negative* flux jumps, as illustrated by dark triangles (ΔB_z_ < 0) in Fig. 5h–i. Here, the negative ΔB_z_ in prior flux filled TVs is accompanied by a partial positive ΔB_z_^p^ in their neighbors (larger brighter triangles near dark triangles in Fig. 5h–i), which is smaller than their regular ΔB_z_ flux jump value. In this case, the flux redistributes by jumping between neighboring TVs due to their magnetostatic coupling assisted by the change in current in the surrounding SC patches. It is different from purely magnetic coupling between electrically insulated SC rings observed in^22^.

**Figure 6:**
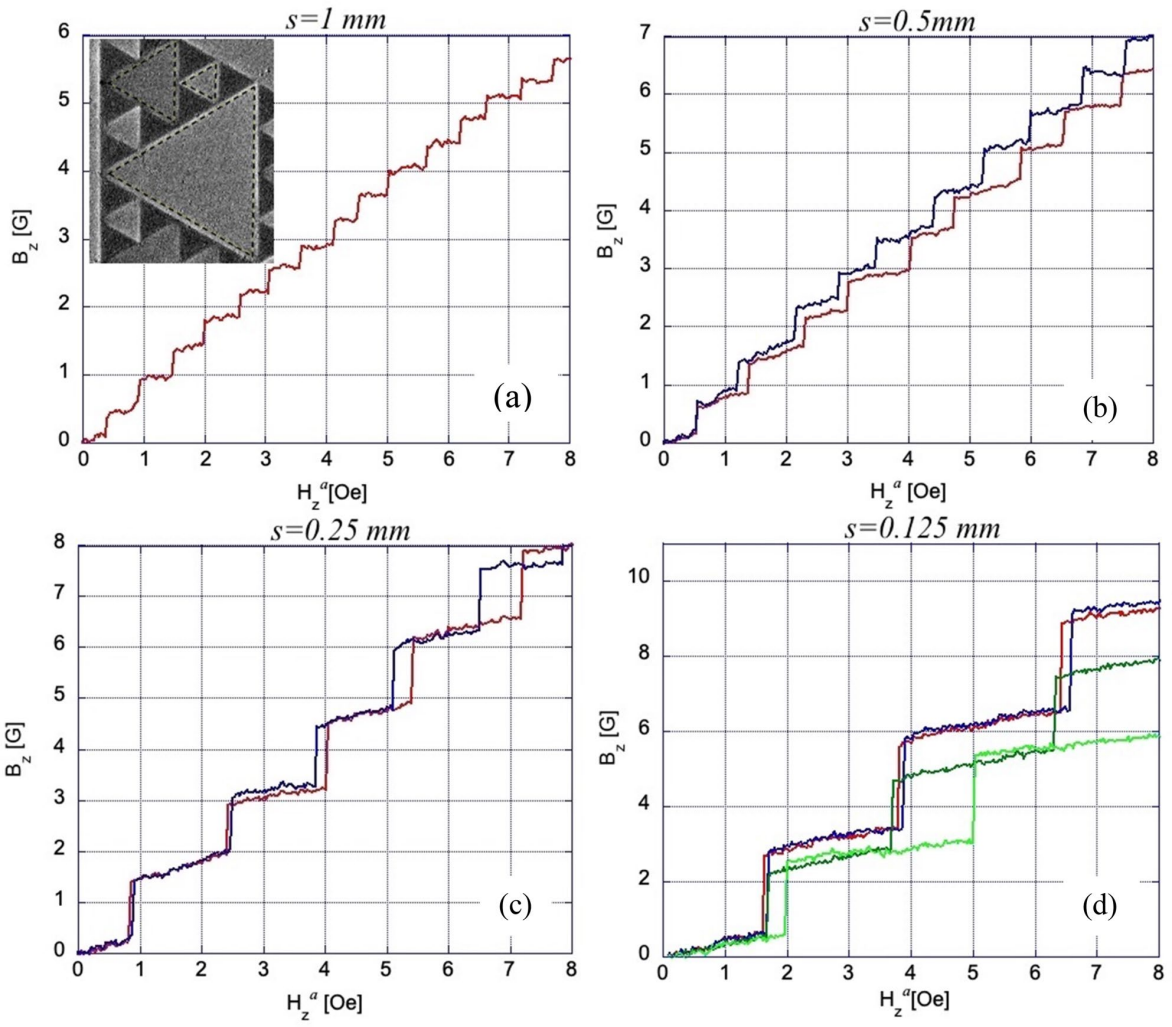
(**a**–**d**) Changes of median normal induction B_z_ in different size triangular voids of the Sierpinski gasket with increasing field H_z_^*a*^ at T = 3.5 K. The insert in (**a**) shows the measurement areas for estimating the median B_z_ in TVs. Successive flux jumps fill the TVs by repeating values of ΔB_z_ in field intervals ΔH_z_^*a*^ which increase with decreasing *s*. B_z_ scales in the plots are different. The jump fields H_z_^*a*^ slightly vary for same size TVs. There is a small difference in ΔB_z_, most noticeable in the smallest TVs, possibly due to imperfections in the narrow bridges between niobium patches. Note the small slope in B_z_(H_z_^*a*^) between the steps as expected in superconducting rings within the London approach.

To quantitatively analyze the magnetic flux evolution in our SG pattern, we measured the MOI signal (I_MOI_) averaged over the area of individual TVs, and transformed I_MOI_ into a median ${\overline{B} }_{z}$ value for the triangle using I_MOI_(B_z_) calibration. Multiplying the obtained ${\overline{B} }_{z}$ by the triangle area we obtain the magnetic flux Φ_i_ acquired by the TV_i_. Figure 6 shows a set of characteristic ${\overline{B} }_{z}($H_z_^ap^) plots for TVs of all four sizes composing the SG. The ${\overline{B} }_{z}$ steps in different TV_i_s are periodic. They have basically the same amplitude and are separated by identical field gaps ΔH_z_^a^ between jumps. The height of the jumps Δ ${\overline{B} }_{z}$ increases with decreasing the TV_i_ size.

There is a slight variation in Δ${\overline{B} }_{z}$ among different triangles of the same size, especially in the smallest TV_4_ (Fig. 6d). This can be due to a small difference of the vertex joints between the SC patches in the structure, which are also responsible for the observed scatter in the first flux jump field for same sized TVs shown in Fig. 3b. Also, the recurrence is disrupted in rare cases of negative or partial flux jumps, when the flux rearranges between neighboring TVs and Δ${\overline{B} }_{z}$ reaches ~ 1/3–1/2 of its regular value (see Fig. A4 in the Supplemental Info).

**Figure 7:**
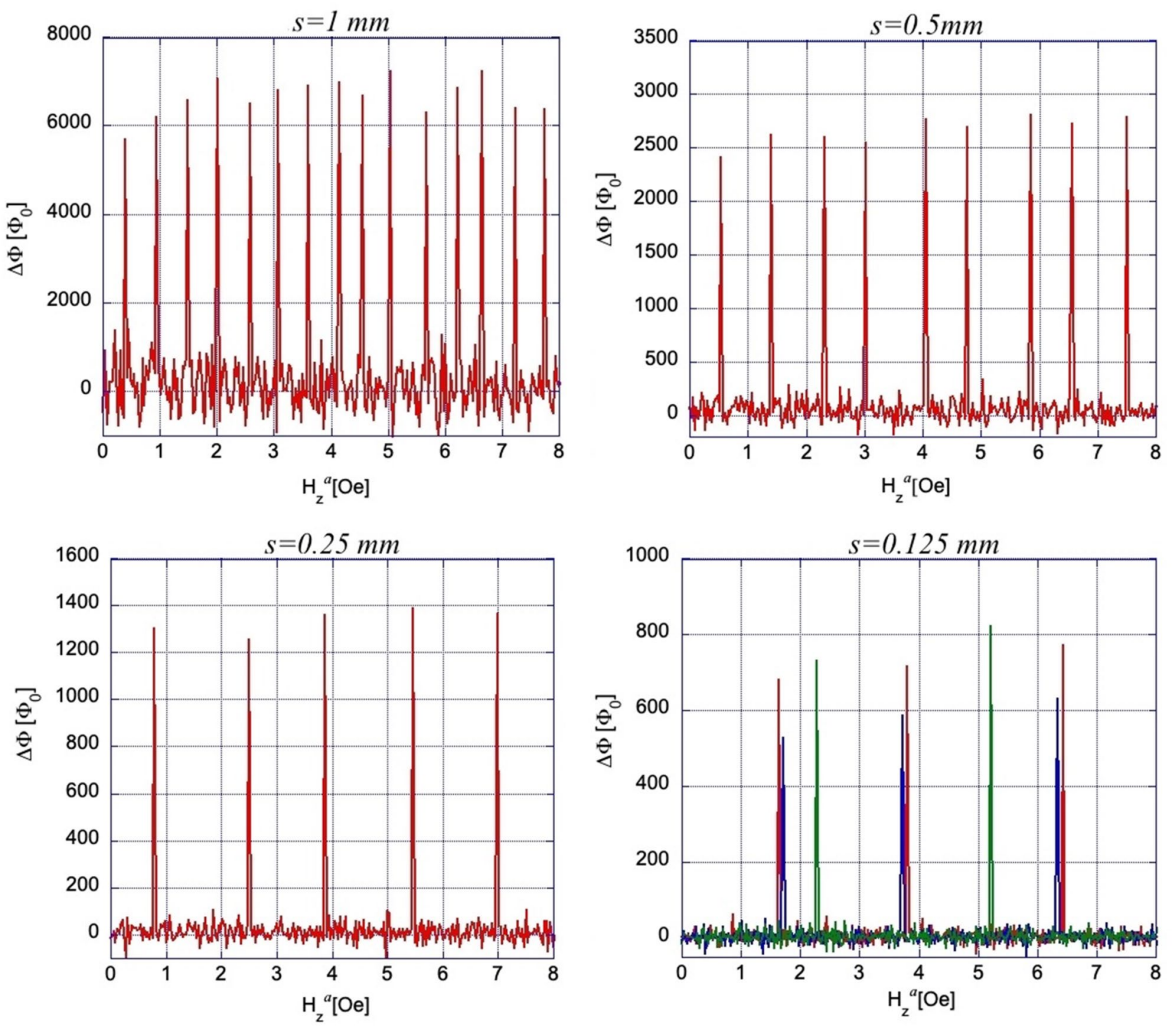
Amplitudes of flux jumps, ΔΦ, in different triangular voids of the Sierpinski gasket at T = 3.5 K. ΔΦ are obtained from measurements of B_z_(H_z_^*a*^) after multiplication by the triangle area. Note different ΔΦ scales in the plots.

**Figure 8:**
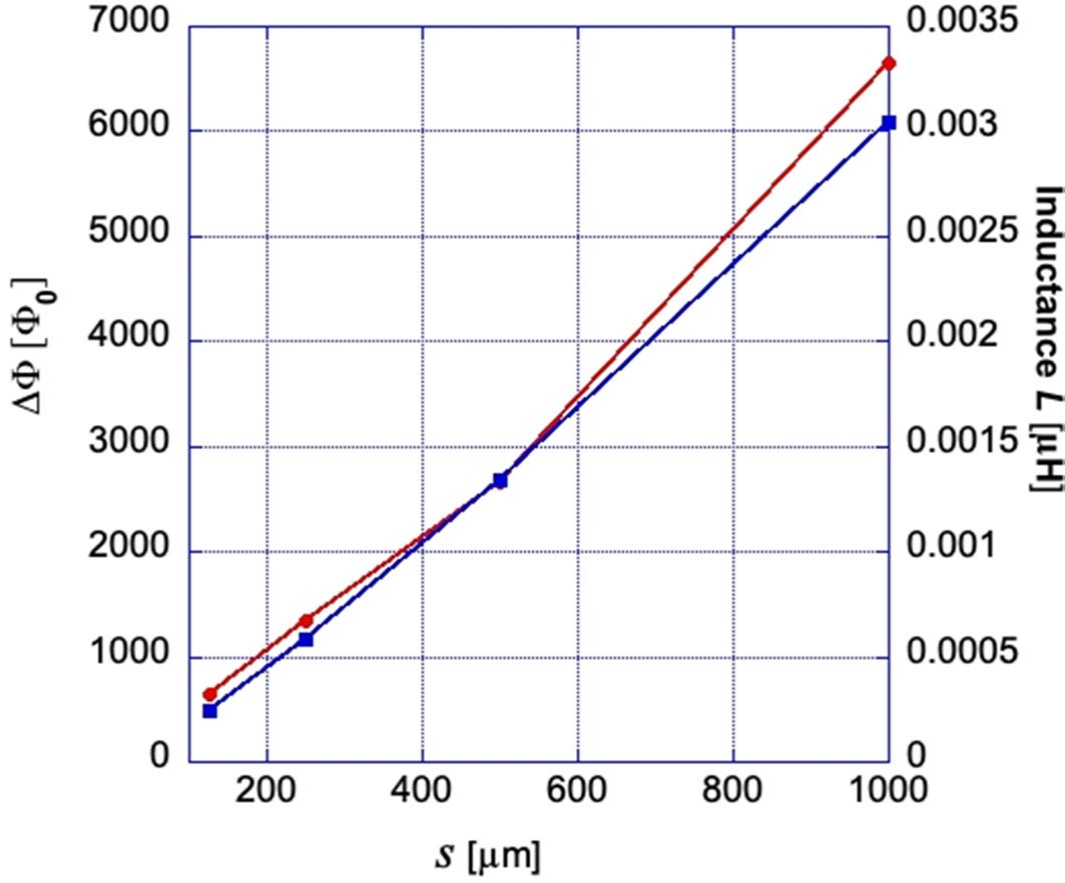
Measured average values of flux jumps ΔΦ in the triangles of different size *s* (red dots) and calculated inductance (blue squares) of narrow rings with geometrically mean radius between circles inscribing and circumscribing the triangle and the same width as the bridge between triangles (see main text).

The distribution of successive flux jump amplitudes ΔΦ_i_ in TV_i_s of different sizes, obtained from Δ${\overline{B} }_{z}$ as those in Fig. 6, is presented in Fig. 7. Here, the average ΔΦ_i_ decreases with *s* from ~ 6600Φ_0_ for the largest TV_1_ to ~ 650Φ_0_ for the smallest TV_4_. Some scatter among successive ΔΦ_i_ in the same TV_i_ is within accuracy of our measurements. Note that the ratio of TV areas S_i_ ~ *s*^2^ in our SG is 1:4:16:64, while ratios of the flux jump values in these TVs (in units of Φ_0_) are ~ 650:1350:2650:6600 (~ 1:2.1:4.1:10.2), i.e. ΔΦ_i_ changes practically linearly with *s* (logΔΦ-log*s* fit gives ΔΦ ~ *s*^1.131^). Assuming that the SC currents, screening the applied field or B_z_ in the TV_i_ due to flux jumps, are concentrated along the sides of the SC triangular patches and at their vertex links, we can model the individual sub-SGs as narrow rings with effective radius *R* = (r_in_R_ci_)^1/2^ = *s*/6^1/2^, intermediate between the inscribed (r_in_) and circumscribed (R_ci_) circles confining the TV_i_. The ring width was chosen as w = 1 µm, corresponding to the width of the bridges between all triangular patches. Appropriate values of inductance *L* of four of our sub-SGs calculated using formula for narrow rings^18^, *L* = µ_0_*R*[ln(8*R*/w) − 2 + ln4], shown by squares in Fig. 8, are consistent with measured mean values of ΔΦ_i_ (round dots) in TVs of different size. This indicates that the inductance of the sub-SGs defines the size of the flux jumps in their central voids.

## Discussion

Experiments on superconducting Sierpinski samples were previously realized on periodic lattices of different order SGs with basic triangles of narrow few-micron long SC nanowires^10–13^ or similar samples containing Josephson junctions in the wires^14,15^. Macroscopic transport and susceptibility measurements on these samples revealed a rich hierarchy of sharp changes of the transition temperature, T_c_(H_z_^*a*^), and inductance, *L*(H_z_^*a*^), corresponding to the complex filling of different size triangles composing the SG with single flux quanta. The theoretical treatment of these results was usually based on the Ginsburg-Landau (GL) equations^13–16,23–25^ assuming the homogeneous magnetic field distribution, i.e. neglecting the SC screening fields. Basically, the superconducting nature of the samples was accounted through the field dependent phase relations of the SC order parameter, which dictate the flux quantization in multiply connected samples. In the case of SG, the flux quanta are predicted to enter the n-order SG with elementary (minimum size) triangles of area A_0_ at fields H > H_c_ = Φ_0_/(4^n^A_0_)^16^. In our SG formed by SC patches, A_0_ is the area of the smallest triangular void, yielding H_c_ ~ (1/4^n^)3 × 10^–3^ Oe, which is much smaller than the observed flux entry fields (~ 0.37 Oe for the 1st flux jump in the central triangle), while the values of flux jumps we measure are much larger than Φ_0_. At the same time, theoretical expectation for successive flux entry, starting from the largest triangle and proceeding to smaller triangles with increasing H_z_^*a*^, is consistent with our observations (compare our Fig. 3 and the diagram of the flux filling sequence in Fig. A5 of Supporting Info, which is plotted using calculations of^16^). However, in our case, the succession of flux entry in different sub-SGs is defined by a distinct mechanism which we discuss below.

Obviously, in our samples at T < T_c_/2 the screening effects are important. Under these conditions, the flux penetration into TVs should occur either through phase slips or by the transit of Abrikosov vortices across the 1 µm bridges connecting the triangular SC patches. Flux penetration occurs when the screening current in these bridges acquires a critical value I_c_. The screening currents flowing over the patches converge in the narrow bridges yielding there the enhanced current density, and with increasing H_z_^*a*^, the total current reaches I_c_ first in these regions. The resulting phase slips or moving vortices temporary suppress the SC order parameter |Ψ| near the vertices of the central TV_i_s in the sub-SGs and provide channels for flux entry. Clearly, the largest current is initially achieved (see Fig. 2a) around vertices of the largest TV_1_ (#1 in Fig. 1) where the first jump occurs. This is then followed by flux jumps into smaller TV_2-4_, and so on, as we observe in our samples.

To understand the regularity and large values of the flux jumps in different TV_i_s in the SG, we presume that the sub-SGs can be considered as inhomogeneous SC islands with a large hole in the center and revisit prior theories of flux quantization in SC rings. For SC rings smaller than the penetration depth λ and with outer radius R of a few ξ, the magnetic field response was widely studied using analytical and numerical solutions of static and time dependent Ginzburg–Landau (TDGL) equations^21,26–39^. These works explained many experimental observations of sharp changes in the microscopic SC ring properties due to the periodic entry of single flux quantum Φ_0_, such as oscillations in T_c_, resistivity, susceptibility, inductance, and heat capacitance^29,30,37,40–44^.

However, computer simulations of TDGL equations^21,31,33^ accounting for different relaxion times of the phase (τ_φ_) and amplitude (τ_|Ψ_|) of the SC order parameter in relatively large rings (R ≳ 10ξ) showed that transitions between many metastable states with different vorticity L_v_ can yield ΔL_v_ >  > 1 (e.g. ΔL_v_ up to 9, i.e. ΔΦ = 9Φ_0_, for R = 15ξ^31^). These transitions repeat at appropriately large field steps (ΔH). They occur if τ_|Ψ|_> > τ_φ_ through phase slips with complicated temporal and spatial variation of φ and |Ψ| depending on the values of relaxation parameters, radius and width of the ring, and ξ, when the gauge-invariant momentum of the SC pairs reaches a critical value p_c_ (i.e. at a critical current)^31,33,35,36^.

In earlier experiments, giant flux jumps with ΔL_max_ = 11 at H_app_ < 40 Oe and gradually decreasing ΔL at larger fields were found in narrow 4µm Al ring at T < T_c_/3^45^. ΔL = 3 jumps were reported for 2µm Al rings^33^. Later, giant flux jump transitions between metastable SC states with ΔL_v_ up-to 70 were detected through sharp changes of the low-T tunnel current in narrow 25 µm square Al rings with a normal electrode in one corner^34,46,47^.

The most intuitive and clear picture of flux quantization in multiply-connected SC samples appears in the London description of the induction and current pattern variations in rings^18–20,48,49^. Unlike the GL formalism, which is mostly applied to mesoscopic rings, the London description is based on electrodynamics equations appropriate for any sample size, while anchoring the flux quantization requirement that maintains the coherent state in the SC material of the ring.

For thin SC rings with dimensions much smaller than the Pearl length (Λ = 2λ^2^/d for ring thickness d << λ), where one can neglect the field induced by the screening currents, the periodic flux entry was explicitly described in^49^. Transitions between states with N and N ± 1 flux quanta in the ring were suggested to occur via the nucleation of Abrikosov (or Pearl for d << λ) vortex or antivortex, either at the outer or inner ring edge, and its motion across the ring width, thus adding or removing one Φ_0_ in the ring annulus. The barrier for this process is defined by the vortex nucleation field. Interestingly, at some fields H ~ (N_1_ + N_2_)/2 the energy for N_1_ and N_2_ states with |N_1_ − N_2_|> > 1 is the same, which could in principle allow large changes of vorticity in the ring.

For large SC rings, where the self-induction contribution becomes important, and where the flux jumps with large vorticity were predicted by the GL calculations, the accurate description of the magnetic response accounting for the self-field induced by the Meissner current was given in^19,20^. The combined solution of Maxwell and London equations showed that the screening currents of the total Meissner state at small applied fields are concentrated near the inner and outer ring edges and have the same sign across the entire ring width. They yield a small induction in the ring annulus, with total flux of less than a flux quantum. At larger H_z_^*a*^, when the flux Φ = NΦ_0_ (N ≥ 1) jumps inside the annulus, the screening current at the inner ring edge changes direction to support Φ, and with further increasing field, the Meissner screening current pattern restores itself until the next flux entry. This picture corresponds to changes of the MOI patterns observed around different voids in our SG samples.

In^20^ Brandt and Clem calculated the SC ring energy in a homogeneous applied field **H**_*a*_ = **B**_*a*_/µ_0_, accounting for the screening currents **j** in the presence of a fluxoid inside the ring and the fields **B**_j_ induced by these currents:

1 $${\text{E}} = \left( {{1}/{2}\mu_{0} } \right)\int {{\mathbf{B}}^{{2}} {\text{d}}^{{3}} {\text{r}}} + (\mu_{0} \lambda^{{2}} /{2})\int {{\mathbf{j}}^{{2}} {\text{d}}^{{3}} {\text{r}}}$$

Here, the first term is the energy of the total field **B** = **B**_*a*_ + **B**_j_ and the second term is the kinetic energy of the currents. The total current and vector potential were divided into parts driven by the fluxoid and by the applied field respectively (details are described in the Supplemental Info). Finally, the Gibbs potential G = E − **mB**_*a*_/2 was obtained, which describes the state of the ring accounting for the **B**_*a*_ source inducing the magnetic moment **m** in the ring. Depending on the applied field, minima of G defined stable flux values in the ring annulus with neighboring states distinct by ± 1Φ_0_.

If we approximate the sub-SG_i_ containing central triangular void TV_i_ with side length *s* as narrow ring with effective radius *R* = (r_in_R_ci_)^1/2^ = *s*/6^1/2^ and follow the same calculations as in^20^ (see Supporting Info S1) we obtain the Gibbs potential responsible for the number, N, of flux quanta in the TV_i_ (omitting the constant homogeneous applied field contribution):

2 $${\text{G}}_{{\text{N}}} = \left( {{1}/\mu_{0} } \right)\left( {{3}/{2}} \right)^{{{1}/{2}}} [{\text{A}}_{{{\text{eff}}}} {\text{B}}_{a} - {\text{ N}}\Phi_{0} ]^{{2}} \left( {{1}/s} \right)/2C$$

Here A_eff_ = π*R*^2^ = (π/6)*s*^2^ is the effective area of the TV_i_ in the sub-SG_i_ and C = [tanh^−1^(*a*/*b*) − 1 + ln4] comes from the inductance of a narrow ring of width w, inner radius *a* = *R* − w/2, and outer radius *b* = *R* + w/2. The minima of G_N_ correspond to the multiquanta states defined by A_eff_ and B_*a*_. However, transitions between different states are delayed until B_*a*_ reaches a characteristic value allowing either phase slips or nucleation and transit of Abrikosov (Pearl) vortices across the narrow bridges in the corners of the TV_i_. These fields are reached when the total screening current in the bridge acquires a critical value I_c_, which yields the flux jump in the TV_i_: Φ = NΦ_0_ = *L*_s-SG_I_c_ (*L*_s-SG_ is the inductance of the sub-SG). After the flux jump, the total current in the bridge vanishes (− j_Φ_ screening the fluxoid inside TV_i_ and + j_H_ screening the applied field compensate each other). With further increasing B_*a*_, j_H_ restores the Meissner distribution over the entire bridge until the total current reaches I_c_ again and an additional fluxoid Φ = NΦ_0_ jumps in. In small fields, as in our experiment, which do not affect the critical current, the jumps should be periodic in field, repeating in steps of ΔB_*a*_ = *L*_s-SG_I_c_/A_eff_.

Similar 1 µm bridges in TV_i_s of all our sub-SGs, should have the same I_c_. However, due to the hierarchical current flow in the entire sample, the critical current is first achieved near the vertices of the largest TV_1_. After the flux enters the largest TV_1_ and the total current through it’s bridges vanishes (∫(j_H_-j_Φ_)dr = 0), the current trajectories form closed loops in the three neighboring smaller sub-SGs and reach I_c_ at their respective TV_i_ bridges with further increasing H_*a*_, resulting in subsequent flux jumps in these TV_i_s. Similar scenario repeats for the next smaller sub-SGs. The flux jumps for smaller structures occur between repeating jumps in larger sub-SGs.

From our data, we can not specify whether the flux jumps in the SG occur due to the phase slips^39,50,51^ or due to the vortex transfer^48^ across the narrow bridge at the vertices. However, quantitative estimates show a faint probability of phase slips in our samples: P ~ exp(− ΔF/k_B_T) with the barrier height ΔF ~ 10^4^ k_B_T_c_ and appropriate critical current density J_c_ ~ 2MA/cm^2^ (see Supporting Info S1). At the same time, J_c_ values obtained from transport measurements of sputtered ~ 100 nm Nb films similar to ours^52^ suggest a high probability of vortex transfer across the SG bridges.

Note, that the succession of giant flux jumps, from largest to smallest sub-SGs, is similar to jump-wise single-Φ_0_ filling of mesoscopic SG numerically calculated within the Ginzburg–Landau approach^16^ (see Fig. A4 in Supporting Info). However, in^16^, where the screening fields are neglected, the flux entry in different sub-SGs is mostly defined by the fluxoid quantization over the sub-SG area in slowly increasing applied field and recurrent current/electric field relations in the SG wire network. In our case, the giant fluxoid entry threshold is defined by the critical current in narrow bridges at the vertices of the sub-SG in the presence of screening effects. Our Gibbs potential analysis models the sub-SGs as independent rings and does not account for their mutual interactions which can be envisioned as magnetostatic coupling between the fluxoids entering different TVs. In our samples we observed a few cases of the flux redistribution between neighboring sub-SGs during separate jumping events (Fig. 5h–i) which are defined by these interactions. However, they were very rare and the individual ring picture seems to capture the main features of the giant flux jumps we imaged.

## Conclusions

In this work, we directly imaged periodic multiquanta magnetic flux jumps in hierarchical fractal-like patterns of superconducting triangular Sierpinski gaskets. Unlike in earlier experiments addressing magnetic oscillations of T_c_ and inductance in Sierpinski structures of microwires or SG networks of Josephson junctions, we studied SG samples of triangular niobium patches with 1 mm to 125 µm sides and directly observed discrete flux filling among proportionally decreasing triangular voids in small perpendicular magnetic fields at low temperatures.

The succession of flux jumps into central triangular voids, TV_i_, of composing the sample sub-gaskets starts with the largest SG and proceeds to sequentially smaller sub-SGs with increasing field. We associate the orderly flux entry into our multiply-connected fractally designed superconducting sample with the controlling role of narrow bridges between continuous SC patches. Here the screening currents converge and with increasing applied field, periodically reach the critical current value, thereby allowing phase-slips or Abrikosov (Pearl) vortex transfer to fill the TV_i_ with multiple flux quanta, NΦ_0_. Considering different sub-SG_i_s, independently, the fluxoid vorticity N is proportional to the inductance *L*_s-SG_ of the sub-SG_i_, which can be approximated by a narrow ring of the order of sub-SG_i_ TV_i_ size *s*, so that N_s-SG_ ~ *s*. In turn, the field periodicity of flux jumps ΔH_*a*_ ~ 1/*s*.

We observe changes of the current patterns during the flux jumps when the screening current around the TV_i_ reverses its direction. The flipped current may compensate the Meissner current induced by H_*a*_ and the total SC current in the TV_i_ joints vanishes, allowing larger current collection at the narrow bridges of smaller TVs and enabling their flux filling. Eventually, multiquanta flux jumps repeat, alternating between large and small sub-SGs where appropriate N_s-SG_ enter at appropriate H_*a*_.

We anticipate that the superconducting Sierpinski structures, where regular giant flux jumps are induced by small applied magnetic fields, may be used for designing low-loss tunable resonators for information and communication technologies. Fine changes in the inductance of the SG pattern due to the controlled fast flux entry in separate sub-SG can allow controlled switching in high frequency operations, in/out signal delivery, and exchange between elements of quantum electronics devices (sensors, amplifiers, memory cells, and computer nodes). The characteristic zero-field frequency response can be adjusted by the SG size and form a wide band of resonance lines depending on the SG order.

## Methods

The samples were fabricated by lift-off procedure of 100 nm niobium film deposited with high vacuum DC magnetron sputtering on a photoresist pattern prepared using laser lithography on a silicon wafer. The accuracy of all 1 µm bridges between triangular patches forming the resulting niobium SGs was inspected in an optical microscope using 100 × objective.

The silicon chips with niobium SG structures were mounted on the cold finger of specially designed optical castle in a helium closed cycle Montana cryostat. A magneto-optical indicator with large Verdet constant was placed on top of the samples, allowing images of the normal magnetic field distributions B_z_(x,y) on their surface in a polarized light microscope. To improve the signal/noise ratio, the magneto-optical images were accumulated using multiple-exposures in a digital 16 bit camera with cooled 1024 × 1024 CCD array. The image intensities I(x,y) were transformed into the B_z_(x,y)-maps using accurate B-I calibration obtained slightly above the superconducting T_c_. Digital operations with images were performed using image processing software.

The description of TDGL simulations of the current distributions in the large Sierpinski gasket without and with magnetic fluxoid in the central triangular void are presented in the Supporting Info, where we also show details of our London calculations of the sub-SG Gibbs potential defining the giant flux jumps in our samples.

### Supplementary Information

Supplementary Information.

## Data Availability

The datasets obtained and/or analyzed during the current study are available from the corresponding author on reasonable request.
