# Supplementary material for: Multiquanta flux jumps in superconducting fractal
Source: Sci Rep. 2023 Aug 3;13:12601. doi: 10.1038/s41598-023-39733-y (PMC10400563; doi:10.1038/s41598-023-39733-y)
Supplement: Supplementary file 1 — Supplementary Information. [file 41598_2023_39733_MOESM1_ESM.pdf]

Multiquanta Flux Jumps in Superconducting Fractal

Vitalii K. Vlasko-Vlasov[1], Ralu Divan[2], Daniel Rosenmann[2], Ulrich Welp[1], Andreas Glatz[1,3] & Wai-Kwong Kwok[1]

[1] Materials Science Division, Argonne National Laboratory, Argonne, Illinois 60439, USA
[2] Center for Nanoscale Materials, Argonne National Laboratory, Argonne, Illinois 60439, USA
[3] Department of Physics, Northern Illinois University, DeKalb, Illinois 60115, USA

A) *Additional Illustrations supporting the main text*

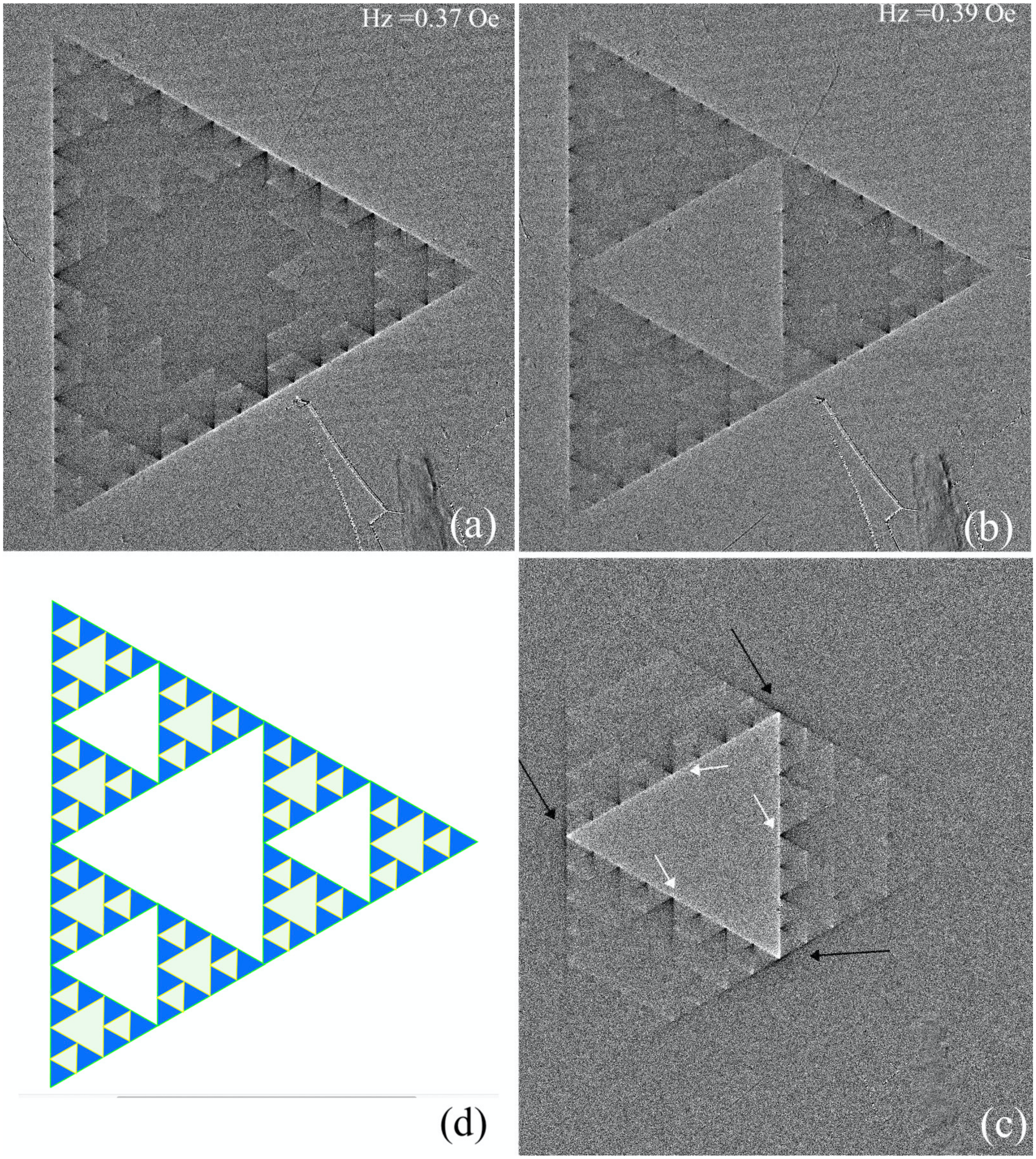

Fig.A1 (a-b) - expanded panels *b-c* of Fig.1 and (c) -expanded panel *a* of Fig.5 of the main text, allowing better view of B_z peculiarities in the vertices of triangular voids (TVs) in the 3d-order

Sierpinski Gasket (SG) before the first flux jump (Meissner state (a)) and after the flux entry in the central TV_1 (b). The difference image in (c) (=(b)-(a)) reveals changes in the current pattern as explained in the main text and illustrated below in Figs.A2-3. It shows the decreased B_z ($\Delta B_z<0$, dark contrast) near the SG outer edges (as indicated by black arrows), where the screening current drops, and the increased B_z ($\Delta B_z>0$, bright contrast) in the central TV_1 and near TV_1 boundaries (pointed by white arrows), where the currents changed the direction to support the entered flux. The in-scale SG pattern with blue triangular niobium patches is shown in (d).

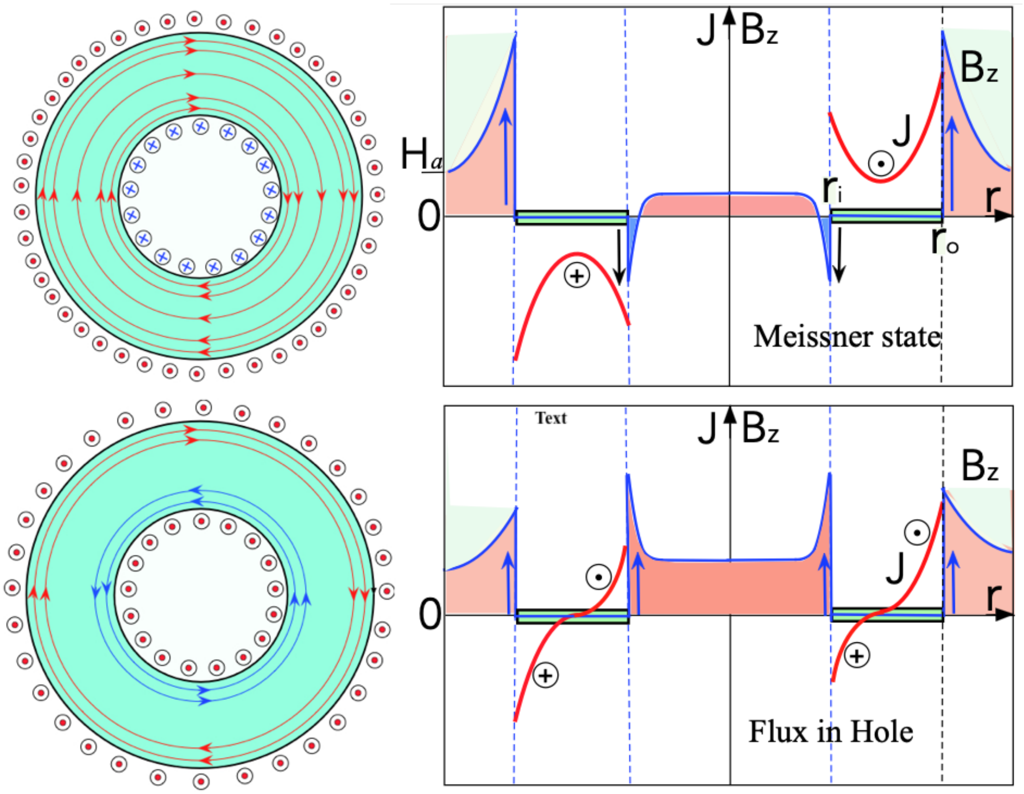

Fig.A2 - Sketch of current (J) and induction (B_z) maps (left panels) in a flat superconducting ring before and after the flux entry into the aperture. Plots of J(r) and B_z(r) are shown on the right (for accurate calculations see [1]). The current (red lines) have the same sign across the ring in the Meissner state (top panels), but inverse direction near the inner ring boundary after the flux entry (bottom panels). J(r) increases towards the ring edges (r_i and r_o) approximately as in a SC strip $J{\sim}r/|r_{i,o}^2 - r^2|^{1/2}$. In the plots, the B_z-polarity is shown by arrows and J-polarity is pointed by small circles with dots and crosses. In left panels, similar circles show Up or Down B_z direction.

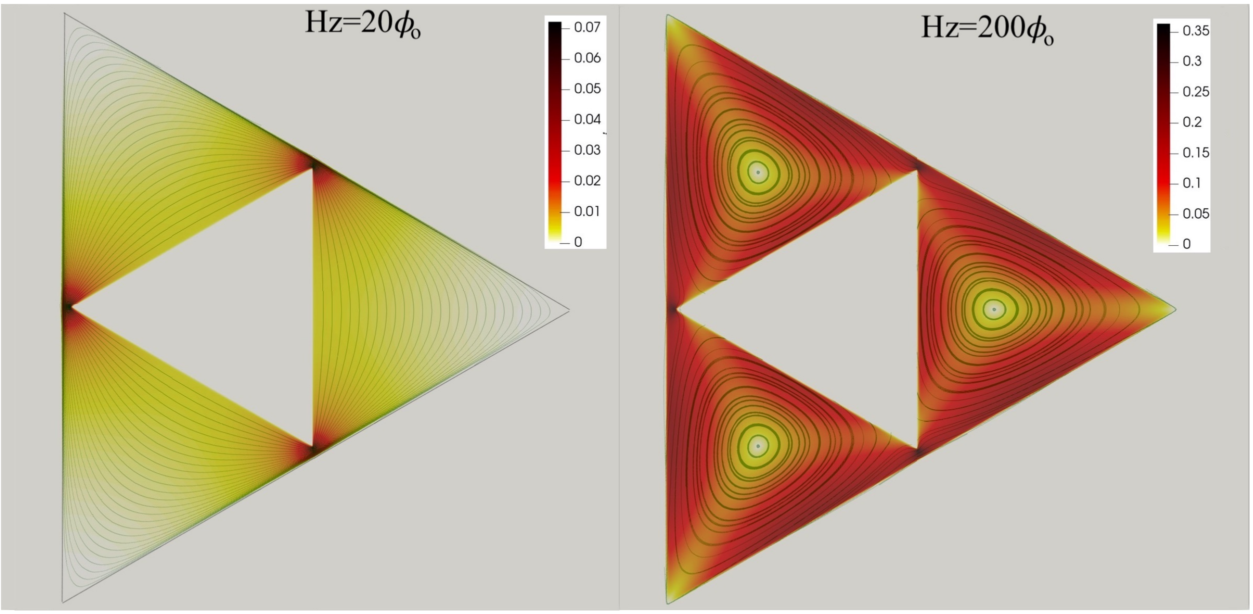

Fig.A3 Current distributions in the Meissner state (applied normal field H_z=20Φ_0) and after the flux entry into the central void (H_z=200Φ_0) of 0-order Sierpiski gasket simulated using the time-dependent Ginzburg-Landau (TDGL) equation. Current lines are superimposed on the current density map in colors of a reduced j_s in units of the depairing current. Note that the current direction along the void boundaries inverts after the flux entry, similar to the case of a superconducting ring sketched in Fig.A2..

The TDGL equation was numerically solved [2, 3] for 0-order superconducting Sierpinski gasket with 500ξ_0 side in 512ξ_0 x 512ξ_0 x 4ξ_0 space. The central equilateral triangular void with 240ξ_0 side leaves 5ξ_0 wide superconducting bridges in the void corners. The void and the space around the SG is modeled as normal material with a ε= (T_c-T)/T= -1 [2]. We use $\kappa=\lambda/\xi>>1$ limit for the solution of the TDGL equations. The system is discretized on a mesh with spacing of $\xi_0/2$. Initially the order parameter is set to a small random number at each grid point and the external magnetic field is set to zero. This system is equilibrated for 1.4x10^6 GL time steps t_0 [2]. Then the z-component of the magnetic field is increased in steps of 20Φ_0/A (A is the x-y area of the system) 10 times. Each field is kept constant for 2x10^5 t_0 steps in order to reach a steady state. From the order parameter distribution in the steady states, we calculate the supercurrent. The flux in the central void (the number of flux quanta) is determined by the phase change of the order parameter along a loop enclosing the void (see [4]).

The current streamlines in the plots are obtained by integration along the supercurrent vectors starting from random seed points. The background shows the magnitude of the supercurrent density.

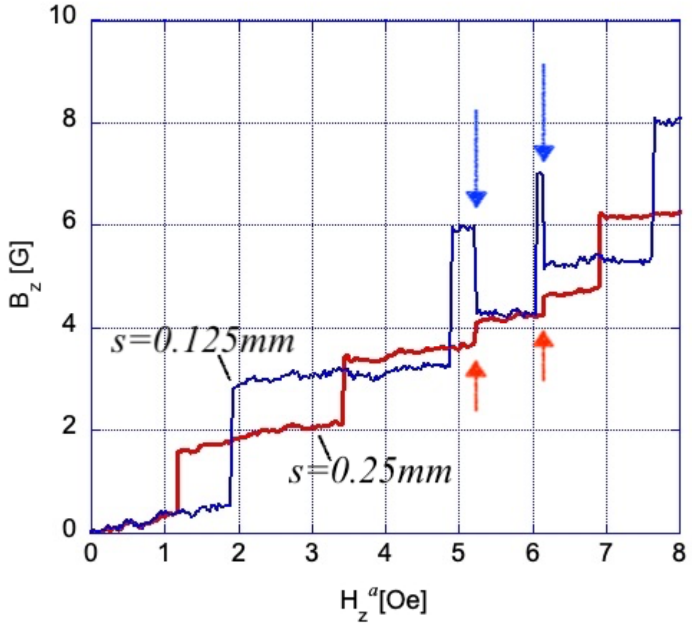

Fig.A4 Changes of B_z during simultaneous negative flux jump (blue arrows) in the smallest triangle (*s*=125µm, blue curve) and partial positive jump (red arrows) in the neighboring larger triangle (*s*=250 µm, red curve). Such jumps are very rare and can be associated with fluctuations. Although similar negative single quantum jumps redistributing flux to the neighboring sub-SGs were found in Ginzburg-Landau simulations of the SG magnetization [5] (see below).

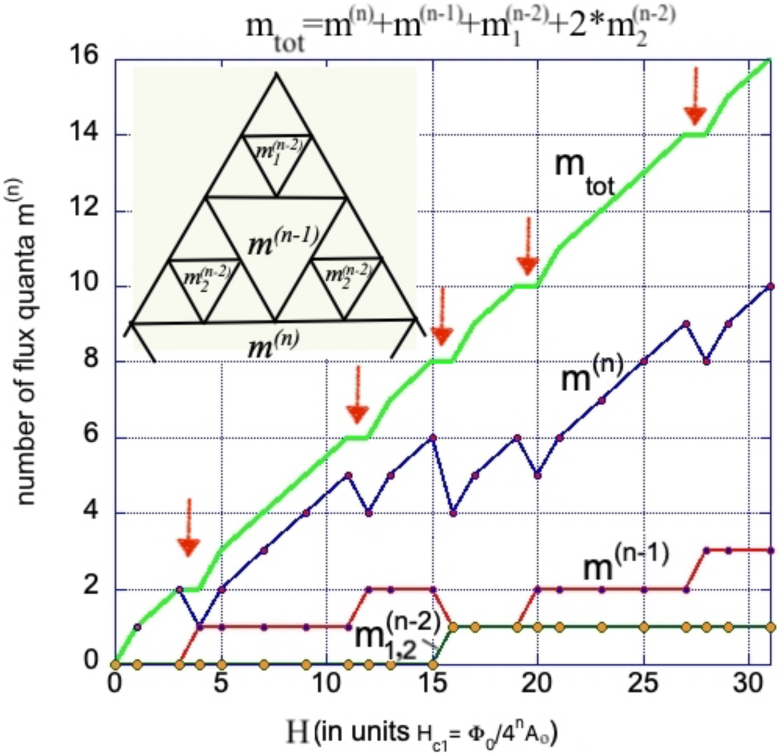

Fig.A5 Diagram of successive filling of nth-order SG, plotted using data of Ginzburg-Landau numerical calculations in [5]. Compare with flux jump succession in Fig.3 of the main text. $m_i^{(n)}$ is

the number of single flux quanta (Φ_0) in triangles appropriately marked in the sketch of SG. The flux enters first in the largest triangle $m^{(n)}$ and then starts filling smaller triangles $m^{(n-1),}$ $m^{(n-2).}$ The symmetric filling in some of them goes in pairs (see formula for m_{tot}). Arrows point to negative jumps in some triangles while positive in their neighbors, so that the total flux m_{tot} does not change but redistributes.

The theory [5] considers the SG made of SC nanowires (see insert), where the magnetic field increasing at a constant rate induces currents **J** and electric fields **E** recurrently changing in the wire network and increasing the SG energy G ~ **E·J** summed over the sample.
The phase relations follow the flux quantization, but the screening fields are ignored, allowing single flux entries inside the triangles at multiples of $\Phi_0/4^n A$, where A is the smallest triangle area and n is the sub-SG order. Numerical minimization of G shows the successive filling of n, (n-1), and (n-2) triangles which goes symmetrically (e.g. in pairs for $m_2^{(n-2)}$). Negative jumps in larger triangles (red arrows) accompany positive jumps in smaller triangles.
The filling succession shown in Fig.A5, although in single Φ_0 steps, is qualitatively similar to multiquanta flux jump observed in our samples (Fig.3b of the main text).

B) *Calculations of the Gibbs potential and macroscopic flux jumps following the London approach developed for flat superconducting rings* in [6].

We start with the total electromagnetic energy of a SC ring in the perpendicular applied field $\mathbf{B}_a = \mu_0 \mathbf{H}_a$ (eq.31 in [6])

$$E = (1/2\mu_0) \int d\mathbf{r}\mathbf{B}^2 + (\mu_0/2)\int d\mathbf{r}\lambda^2\mathbf{j}^2 \qquad \text{(B1)}$$

Here $\mathbf{B}=\mathbf{B}_a+\mathbf{B}_J$ includes the field of screening currents $\mathbf{B}_J$, the first ∫ over the whole space is the field energy, and the second ∫ over the ring is the kinetic energy of SC currents.
Using relations: $\mathbf{B}=\mu_0\mathbf{H}$, $\mathbf{B}=\nabla\times\mathbf{A}$, $\mathbf{j}=\nabla\times\mathbf{H}=\nabla\times\mathbf{B}/\mu_0$, $\mathbf{B}^2=\mathbf{B}\nabla\times\mathbf{A}$, $\nabla\times\mathbf{B}_a=0$, the 1st integral in (B1) is:

$$\int \mathbf{B}^2\, d\mathbf{r} = \int (\mathbf{B}_a^2 + 2\mathbf{B}_a\mathbf{B}_J + \mathbf{B}_J^2)\, d\mathbf{r} = 2\mu_0 E_a + 2\int \mathbf{B}_a\nabla\times\mathbf{A}_J\, d\mathbf{r} + \int (\mathbf{B}_J\nabla\times\mathbf{A}_J) d\mathbf{r} =$$
$$= 2\mu_0 E_a + 2\int\nabla\times\mathbf{B}_a\mathbf{A}_J\, d\mathbf{r} + \int\nabla\times\mathbf{B}_J\mathbf{A}_J\, d\mathbf{r} = 2\mu_0 E_a + \int\mu_0\mathbf{j}_J\mathbf{A}_J\, d\mathbf{r} = 2\mu_0 E_a + \int\mu_0\mathbf{J}_J\mathbf{A}_J\, d^2\mathbf{r} \qquad \text{(B2)}$$

with $2\mu_0 E_a = \int \mathbf{B}_a^2\, d\mathbf{r}$.

Brandt and Clem [6] divide fields and currents into two parts – 1- driven by *fluxoid*, and 2- driven by the *applied field*:
$\mathbf{B}=\mathbf{B}_1+\mathbf{B}_2$, $\mathbf{A}=\mathbf{A}_1+\mathbf{A}_2$, $\mathbf{j}=\mathbf{j}_1+\mathbf{j}_2$, $\mathbf{J}=\mathbf{J}_1+\mathbf{J}_2$ ($\mathbf{J}=\mathbf{j}d$ in d-thick ring).
Near the ring plane, z=0, for the homogeneous applied field $\mathbf{B}_a$=const, the vector potential $A_a(r) = rB_a/2$. In turn, for two components of the current (J_1 and J_2), the London eq-ns are :

$$J_1(r) = -[-\Phi/2\pi r + A_{J1}(r)]/\mu_0\Lambda \ \ (\text{with } \Lambda=\lambda^2/d) \rightarrow \ A_{J1}(r) = -\mu_0\Lambda J_1(r) + \Phi/2\pi r$$
$$J_2(r) = -[A_a(r) + A_{J2}(r)]/\mu_0\Lambda \qquad \rightarrow \ A_{J2}(r) = -\mu_0\Lambda J_2(r) - rB_a/2$$

Using these components, $\mathbf{J}_J\mathbf{A}_J$ in (B2) becomes:

$$(J_1+J_2)(A_{J1}+A_{J2}) = J(-\mu_0\Lambda J_1(r) + \Phi/2\pi r - \mu_0\Lambda J_2(r) - rB_a/2) = J(-\mu_0\Lambda J + \Phi/2\pi r - rB_a/2) =$$
$$= -\mu_0\Lambda J^2 + J(\Phi/2\pi r - rB_a/2) \qquad \text{(B3)}$$

Here the 1st term compensates the second integral in (B1).

Finally, using the magnetic moment **m** of the ring m= ∫dr πr^2 J(r), and the total current I=∫dr J(r), after integration of (B3) with 2πrdr over the ring, and accounting for the factor $1/2\mu_0$ and the fluxoid quantization $\Phi = N\Phi_0$, E in (B1) reduces to [6]:

$$E = E_a + \mathbf{mB}_a/2 + N\Phi_0 I/2 \qquad \text{(B4)}$$

Then the Gibbs potential G=E-$\mathbf{mB}_a$, which describes the system in the presence of $\mathbf{B}_a$=const, and accounts for the work -$\mathbf{mB}_a$ of external sources inducing the magnetic moment **m**, is:

$$G = E_a - \mathbf{mB}_a/2 + N\Phi_0 I/2 \qquad \text{(B5)}$$

Now we admit that the main screening currents in a sub-SG like that in Fig.A2 are concentrated near the edges of the SC triangles and are maximum in the connecting bridges. Accounting that the strongest field contributions come from the currents flowing around the boundaries of the central empty triangle and the largest currents along the bridges in it's vertices, we approximate the sub-SG with a ring of a geometric mean radius between the inscribed and circumscribed circles $\mathcal{R}=(rR)^{1/2} = s/6^{1/2}$. The ring width is taken that of the bridges, w=1μm.

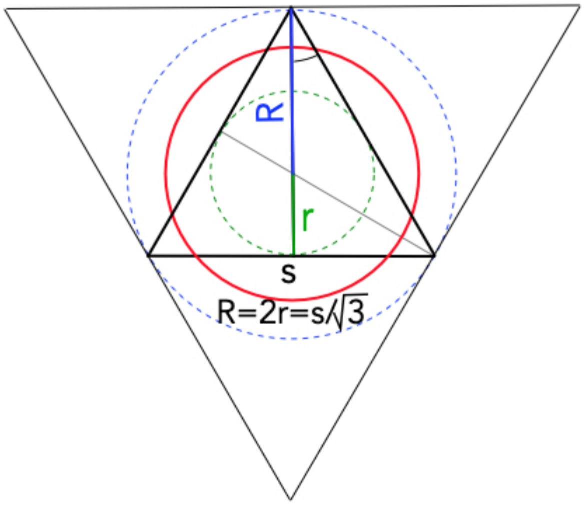

Fig.A6 Choice of effective radius.

Using the inductance of a narrow ring, $L=\mu_0\mathcal{R}[\ln(8\mathcal{R}/w)-2+\ln 4]$ $(\equiv 2\mu_0\mathcal{R}[\tanh^{-1}[(2\mathcal{R}-w)/(2\mathcal{R}+w)]-1+\ln 4])$, and the flux which compensates the external field, $\pi\mathcal{R}^2 B_a = -LI_2$, one finds the magnetic moment due to B_a : $m_2=\pi\mathcal{R}^2 I_2 = -(\pi\mathcal{R}^2)^2 B_a/L$. The magnetic moment induced by the fluxoid, Φ, is $m_1=\pi\mathcal{R}^2 I_1$ with $I_1=\Phi/L$. Note, that the formula for L we use, suggests a constant current density in the ring, which is expected in the bridges of sub-SG (J_c -critical), when the flux jumps into appropriate TV. More accurate account of the current distribution triangular patches around the TV should not essentially change nearly linear dependence of L on the sub-SG size ($L \sim \mathcal{R} \sim s$).

After substitution of $m=m_1+m_2$ and $I=I_1+I_2$ in (B5) the Gibbs energy for a sub-SG with the central empty triangle side s (approximated by a ring) becomes:

$$G_N = (1/\mu_0)(3/6^{1/2})[A_{eff}B_a - N\Phi_0]^2(1/s)/2C \qquad \text{(B6)}$$

Here $C = (1/2)\,[\ln(8\mathcal{R}/w)-2+\ln 4]$ is a factor weakly dependent on s, and $A_{eff} = (\pi/6)s^2$ is the area of the effective ring opening. The permanent applied field energy $\sim B_a^2$ is omitted in (B6).

According to our model, the flux jumps occur when the screening current in the bridges between the SC patches in the apexes of the central empty triangle of the sub-SG, acquires the critical value I_c (critical density J_c across the bridge). Then the applied field steps between the flux jumps in the sub-SG are $\Delta B_a = L I_c / A_{eff}$ and corresponding values of the subsequent giant flux jumps are $N\Phi_0 = A_{eff}\Delta B_a = L I_c$.

C) *Estimates of probability of the phase slips in the links between SC patches.*

To estimate the probability of phase slips in our samples we use the developed theoretical approach to this effect [7, 8, 9]. It is known that during the phase slip, an L-long and coherence length (ξ) wide normal channel is formed for a very short time $\sim\tau_{GL}$ [10]. It interrupts the persistent current flow and may allow the field entry inside a void. The energy barrier of the phase slips, $\Delta F(T) = (\sqrt{6}/2\pi)\, \Phi_0 I_c(T)$, is defined by the critical current I_c . In turn, their probability is $P\sim \exp(-\Delta F/k_B T)$ if they are thermally activated, or $P\sim\exp(-\Delta F/k_B T_c)$ if they appear due to the macroscopic quantum tunneling [8]. Following [8], the low temperature critical current $I_c(0)=(92\mu A)(T_c/R_n)(L/\xi(0))$, where R_n is the normal resistance of the slip channel in Ω and T_c is in ^{o}K. For the channel length L , which in our case is the bridge width w=1μm, film thickness d=0.1μm, channel width ξ ($\xi(0)\sim$20nm in our samples [11]), and normal resistivity $\rho_{Nb}(10K)\sim 4\mu\Omega\cdot cm$, we have $R_n=\rho_{Nb} w/d\xi \sim 20\ \Omega$ and $I_c\sim$ 2 mA for our SG vertex bridges. As a result, $\Delta F/k_B T_c \sim 10^4$ for $T_c\sim$9K , which makes the feasibility of the phase slips unrealistic, even with a large preexponential factor of the attempt frequency $\sim 1/\tau_{GL}$. In our case, $\tau_{GL}=4\pi\sigma_n\lambda^2/c^2 \sim 2.2\times10^{-12}$ sec (for $\sigma_n=1/\rho_{Nb}$, λ=84nm [11], and speed of light c). At the same time, appropriate current density, $J_c= I_c/wd \sim 2$ MA/cm^2, which is close to J_c associated with vortex dynamics in sputtered ~100 nm Nb films (e.g.[12]), suggests a high probability of the vortex transfer across the bridges that could deliver the multiquanta flux in the SG triangular voids.

References

[1] A. A. B. Brojeny, J. R. Clem, ***Magnetic-field and current-density distributions in thin-film superconducting rings and disks***, Phys. Rev. B **68**, 174514 (2003).

[2] A. Sadovskyy, A. E. Koshelev, C. L. Phillips, D. A. Karpeev, and A. Glatz, ***Stable large-scale solver for Ginzburg-Landau equations for superconductors***, J. Comp. Phys. **294**, 639 (2015).

[3] W.-K. Kwok, U. Welp, A. Glatz, A. E. Koshelev, K. J. Kihlstrom, G. W. Crabtree, ***Vortices in High-Performance High-Temperature Superconductors***, Rep. Prog. Phys.**79**, 116501 (2016).

[4] C. L. Phillips, T. Peterka, D. Karpeyev, and A. Glatz, ***Detecting vortices in superconductors: Extracting one-dimensional topological singularities from a discretized complex scalar field***, Phys. Rev. E **91**, 023311 (2015).

[5] A. Ceccatto, S. Doniach, K. Frahm, and B. Miihlschlegel, ***The nature of the flux lattice in granular superconducting networks***, Z. Phys. B **82**, 257 (1991).

[6] E. H. Brandt, J. R. Clem ***Superconducting thin rings with finite penetration depth***, Phys. Rev. B **69**, 184509 (2004).

[7] D. E. McCumber and B. I. Halperin, ***Time scale of intrinsic resistive fluctuations in thin superconducting wires***, Phys. Rev.B **1**, 1054 (1970).

[8] M. Tinkham and C. N. Lau, ***Quantum limit to phase coherence in thin superconducting wires***, Appl. Phys. Lett. **80**, 2946 (2002).

[9] G. P. Papari and V. M. Fomin, ***Quantum interference in finite-size mesoscopic rings***, Phys. Rev. B **105**, 144511 (2022).

[10] M. Tinkham, ***Introduction to Superconductivity***, Courier, New York, NY (1996).

[11] V. K. Vlasko-Vlasov, F. Colauto, A. A. Buzdin, D. Carmo, A. M. H. Andrade, A. A. M. Oliveira, W. A. Ortiz, D. Rosenmann, and W.-K. Kwok, ***Crossing fields in thin films of isotropic superconductors***, Phys. Rev. B **94**, 184502 (2016).

[12] I. V. Yanilkin, A. I. Gumarov, A. M. Rogov, R. V. Yusupov, and L. R. Tagirov, ***Synthesis of Thin Niobium Films on Silicon and Study of Their Superconducting Properties in the Dimensional Crossover Region***, Techn. Phys. **66**, 263 (2021).
